# Study on the relationship between intrapartum group B streptococcus prophylaxis and food allergy in children

**DOI:** 10.3389/fped.2022.1039900

**Published:** 2022-12-02

**Authors:** Hong Zhang, Kang Xu, Zhihui Liu, Yuanmei Shi, Hui Li, Xiaoping Yin

**Affiliations:** ^1^ Department of Pediatrics,Taixing People's Hospital, Taizhou, Jiangsu, China; ^2^ School of Clinical Medicine, Bengbu Medical College, Bengbu, Anhui, China

**Keywords:** group b streptococcus, intrapartum antibiotic prophylaxis, food allergy, group b streptococcus, children

## Abstract

**Objective:**

To investigate the associations between intrapartum antibiotic prophylaxis of group B streptococcus (GBS) in pregnant women and the risk of food allergy in Chinese children

**Design:**

Retrospective cohort study of 2,909 mother-child pairs.

**Setting:**

Taixing People's Hospital in Eastern China.

**Participants:**

Term infants born 2018–2019, followed longitudinally from birth to 3 years.

**Exposures:**

The GBS-IAP was defined as therapy with intravenous penicillin G or ampicillin or cefazolin ≥4 h prior to delivery to the mother. Reference infants were defined as born without or with other intrapartum antibiotic exposure.

**Methods:**

To investigate the incidence information of food allergy in children aged 18 months and three years old. Kaplan-Meier survival analysis and log-rank tests were used to evaluate the cumulative incidence in the group with GBS-IAP and the group without GBS-IAP. Cox proportional hazards models were conducted to determine the univariate and multivariate association between maternal GBS-IAP and incident food allergy after various covariates were adjusted.

**Results:**

The cumulative incidence of food allergy in the group with GBS-IAP was higher than that in the group without GBS-IAP in children under 18 months old (8.1% vs. 4.5%, *P* = 0.005, log-rank test), but no significant differences were observed in children under three years old (9.2% vs. 7.0%, *P* = 0.146, log-rank test). The univariate cox proportional hazards model in children under 18 months old revealed that children in the GBS-IAP group had faster food allergy development when compared with children in the group without GBS-IAP (HR.: 1.887,95% CI: 1.207–2.950, *P* = 0.005), so was the multivariate model (HR.: 1.906,95% CI: 1.158–3.137, *P* = 0.011). However, both univariate (HR: 1.343, 95% CI: 0.891∼2.026, *P* = 0.159) and multivariate (HR: 1.253, 95%CI: 0.796∼1.972, *P* = 0.329) cox proportional hazards model in children under three years old showed no significant differences between children in the group with GBS-IAP and group without GBS-IAP.

**Conclusion:**

Intrapartum antibiotic prophylaxis of group B streptococcus may increase the cumulative incidence and risk of food allergy in children under 18 months old, but it had no significant effect on children under three years old.

## Introduction

Food allergy is a worldwide public health issue, especially in children (1, 2). Recent research showed that a total of 1.5 million Americans, including 4% of children and 1% adults, suffered from food allergies (3, 4). In China, the incidence of food allergy is gradually approaching that of western countries. For example, 3.5% to 7.7% of children under two years old in Chongqing in 2012 were reported to develop food allergies (5). The continuous growth of the prevalence of food allergy in children not only significantly impacts physical health but also contributes to a considerable burden on the medical system.

Some studies suggested that antibiotic exposure in early life may increase the risk of allergic diseases in children (6–8). Still, such studies mainly focus on the use of antibiotics in the early postnatal period of children, and few studies explored the long-term effect of antibiotics used in the critical period before delivery. Group B streptococcus (GBS), a Gram-positive and facultative anaerobic bacterium, inhabits the gastrointestinal and urogenital system of about 18% of pregnant women worldwide (9). Vertical transmission and colonization during delivery is the most significant risk factor for early-onset disease (GBS-OED) in newborns (10, 11). Since the mortality related to EOD is as high as 1% to 3%, intrapartum antibiotic prophylaxis (IAP) is mainly used to prevent GBS-OED at present (11). IAP was first introduced to avoid GBS infection in the 1990s, and the long-term side effects of antibiotics were not well studied. Nevertheless, clinical studies have reported that maternal use of antibiotics during pregnancy is related to the increased risk of childhood atopic diseases such as food allergy in recent years (12).

In this study, we recruited 2,909 full-term non-infected newborns to explore whether GBS-IAP was associated with the increased risk of food allergy in children, especially children under three years old. With this manuscript, we would like to provide a theoretical basis of the association between GBS-IAP and subsequent food allergy in children, and then promote the reasonable application of GBS-IAP.

## Materials and methods

### Study sample

Pregnant women and full-term newborns delivered in Taixing People's Hospital from January 2018 to February 2019 were recruited. Inclusion criteria were full-term infants with a gestational age ≥37 weeks and a birth weight ≥2,500 g. Exclusion criteria included (1) Maternal age <18 years; (2) No informed consents signed; (3) Residence duration in Taixing <6 months; (4) Parents without the ability to communicate; (5) Parents with neuropsychiatric disorders; (6) Neonatal sepsis within 72 h after delivery. A total of 3,500 mother-child pairs were enrolled in this study initially, and 320 pairs were excluded because of miscarriage and induced abortion. 71 pairs were excluded due to abnormal development and chronic disease, and 200 pairs were excluded because of loss of follow-up and incomplete information. To be more specific, 11, 20 and 28 mother-child pairs were excluded due to missing information about maternal history of allergic diseases, maternal education level and maternal smoking history, respectively. Finally, 2,909 mother-child pairs were adopted as the final sample (Figure 2). The study was approved by the Ethics Committees of Taixing People's Hospital (Project number: txry2018-003) in Jiangsu Province, China.

**Figure 1:**
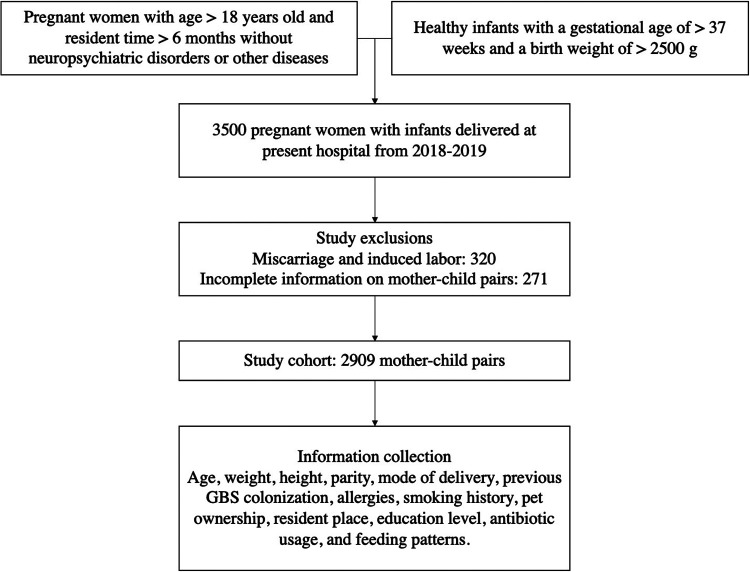
Design of study cohorts. Figure describes the process of inclusion and exclusion criteria for the selected pregnant women and infants. GBS, group B Streptococcus; IAP, intrapartum antibiotic prophylaxis.

**Figure 2:**
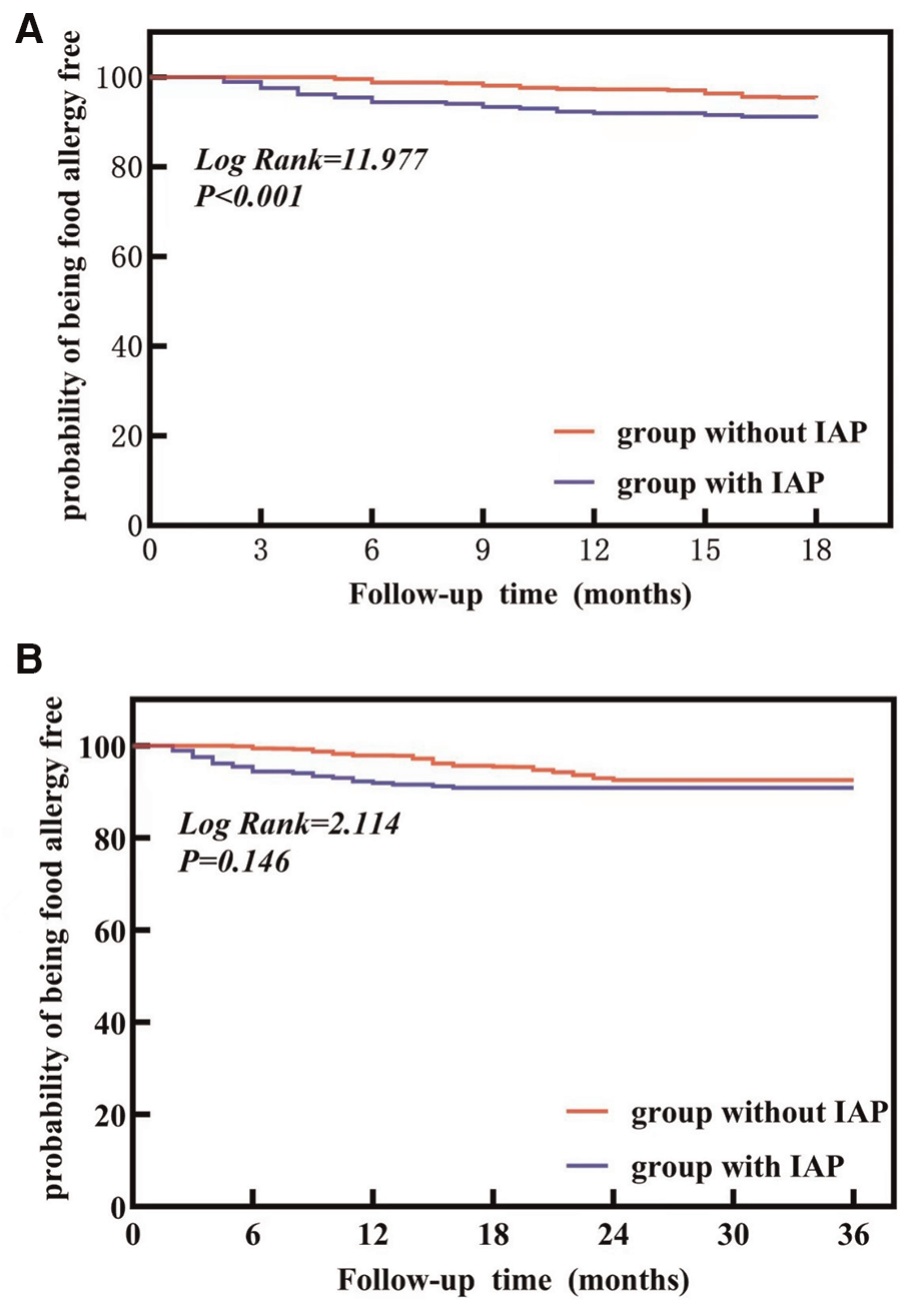
The survival curve for the development of food allergy according to Intrapartum Antibiotic Prophylaxis (IAP) (**A**) represents a follow-up of 18 months. (**B**) represents followed-up to 3 years old. IAP, intrapartum antibiotic prophylaxis.

### Information collection

The hospital information system was employed to collect maternal information about age, body weight, height, parity, mode of delivery, GBS colonization, and IAP, and so was the news about gestational age (recorded as weeks), gender, birth weight (recorded as gram), antibiotic use within 72 h after birth in newborns. Questionnaires were adopted to collect information about the maternal smoking history, pet keeping and allergic diseases, maternal education level, and feeding patterns.

### Exposure

The primary exposure was maternal intrapartum GBS prophylaxis (GBS-IAP) defined as intravenous penicillin, ampicillin, cefazolin, or clindamycin administered within 4 h before delivery (13, 14). All the pregnant women of positive GBS screening in this study received the IAP as recommended by national guidelines during the study period. All other forms of maternal antibiotic therapy were classified as “no GBS-IAP” antibiotic exposure, including non GBS-specific antibiotics, GBS-specific antibiotics administered <4 h before delivery, and surgical prophylaxis administered to women undergoing cesarean delivery.

### Outcome

The study was based on the EuroPrevall program of the international study of asthma and allergies in childhood (ISAAC) (15), Initial screening questionnaires were distributed to all parents or guardians after face-to-fece interviews to investigate information about food allergy in children before 18 months old and 3 years old from February to March in 2022. The preliminary screening questionnaire included relatively simple questions, such as “whether children were diagnosed with food allergy by professional doctors?”. Parents would be asked to complete a detailed standardized written detailed questionnaire, if the initial screening questionnaire reported the occurrence of food allergy. Besides, we provided participants with free medical physical examination and detailed medical suggestions to increase the efficient response rate. The standardized detailed questionnaire (Version number: OCT07VERSION) was derived from the EuroPrevall program (15), and it involved multiple questions as followed: maternal history of allergic diseases, infant feeding patterns, and maternal smoking history, etc (see supplementary materials).

Accordingly, their children were examined by specialists, and the diagnostic criteria of food allergy were referred to the consensus of experts on the prevention, diagnosis, and treatment of infant allergic diseases, issued by the Immune Groups, The Society of Pediatrics, Chinese Medical Association (16).

The diagnostic criteria are briefly described as follows: 2460;. Detailed medical history and physical examination, at least one of the following food allergy symptoms: poor sleep, crying, restlessness, rash, frequent nasal itching, rubbing eyes, runny nose, sneezing, wheezing, vomiting, diarrhea, constipation, constipation. ②.the clinical allergic symptoms were relieved or disappeared after avoiding suspicious allergic foods and aggravated or reappeared after reintroducing suspect foods; ③ positive serum-specific IgE test or skin prick test;. 2463;. Positive food provocation test. Having ①, plus any of ② ③.④, would be diagnosed as food allergy.

### Covariates

Based on previous studies (17–21), maternal age, maternal BMI, maternal history of allergic diseases, and maternal smoking history were included as covariates, and so were gestational age, gender, birth weight, and Parity. Besides, behavioral factors including keeping pets, feeding patterns, and antibiotic use within 72 h after birth in newborns were included in the multivariable logistic regression model since they were reported to be associated with food allergy in children.

Maternal smoking history was defined as smoking during pregnancy and after delivery. Maternal history of allergic diseases was defined as the diagnosis of asthma, atopic dermatitis, or food allergy. Feeding patterns include breastfeeding (only breastfeeding was adopted within six months), artificial feeding (no breastfeeding was adopted within six months), and mixed feeding (both breastfeeding and powdered milk were adopted).

### Quality control

The questionnaires were distributed and explained by well-trained investigators, and questionnaires were numbered in sequence during the on-site investigation. After parents finished the questionnaire, the contents of the questionnaires were double-checked and proven to be complete. Each questionnaire was double entered to reduce entry errors, and the response rate was 98% after quality control.

### Statistical analysis

Initially, chi-square tests, one-way ANOVA and Kruskal-Wallis test were used to compare the baseline characteristics between a group with GBS-IAP and a group without GBS-IAP. Then Kaplan-Meier survival analysis and log-rank tests were used to evaluate the cumulative incidence in the group with GBS-IAP and the group without GBS-IAP. Finally, cox proportional hazards models were conducted to determine the univariate and multivariate association between maternal GBS-IAP and incident food allergy after various covariates were adjusted. Information in the hospital information system and questionnaires was input with Epidata3.0, and statistical analyses were employed in SPSS26.0, while forest plots were drawn with R 4.1.3. Two-sided tests were conducted with an inspection level set at *α *= 0.05.

## Results

### Baseline characteristics

As shown in Table 1, 2,909 mother-child pairs were included in the final sample. A total of 368 pregnant women were screened positive for GBS with a colonization rate of 12.6%, while 284 (9.8%) received GBS-IAP. Statistically significant differences between group with IAP and group without IAP were detected, including mode of delivery, feeding patterns, maternal history of allergic diseases, maternal smoking history, keeping pets, antibiotics usage within 72 h after birth, gestational age, and birth weight. To be more specific, children in the group with GBS-IAP were more likely to receive breastfeeding (82.4% vs. 77.0%, *P *= 0.018), and antibiotic use within 72 h after birth (47.1% vs. 8.0%, *P *< 0.001), when compared with children in the group without GBS-IAP. Besides, mothers in the group with GBS-IAP were more likely to have a history of allergic diseases (13.7% vs. 8.6%, *P *= 0.004) and smoking (15.1% vs. 9.7%, *P *= 0.004), but the ratio of keeping pets (26.1 vs. 32.3%, *P *= 0.032) and cesarean section (42.3% vs. 48.7%, *P *= 0.038) was lower than that of the group without GBS-IAP.

**Table 1:** Demographic and other characteristics of study subjects.

Characteristic	Without GBS- IAP (*n* = 2625)	With GBS- IAP (*n* = 284)	*t*/*χ* **2**	*P value*
Mother's age (years)	28.15 ± 4.23	28.18 ± 4.35	−0.120a	0.905
Mother's BMI (x ± s)	20.90 ± 1.73	20.99 ± 1.68	−0.899 a	0.369
Parity (*n*, %)			0.429 b	0.512
One child	1,320 (50.2)	137 (48.2)		
Second child or more	1,305 (49.8)	147 (51.8)		
GBS Screening (*n*, %)			256.132 b	<0.001
Positive	98 (3.7)	270 (95.1)		
Negative	2,527 (96.3)	14 (4.9)		
Mother's allergy history (*n*, %)	225 (8.6)	39 (13.7)	8.272 b	0.004
Whether received higher education (*n*, %)	2,003 (76.3)	223 (78.5)	0.701 b	0.403
Gender (man, *n*, %)	1,413 (53.8)	161 (56.7)	0.845 b	0.358
Gestational age (weeks x ± s)	39.4 ± 1.0	39.2 ± 1.0	2.517 a	0.012
Weight of birth (g x ± s)	3411 ± 425	3469 ± 444	−2.186 a	0.029
Feeding method (*n*, %)			8.057 b	0.018
Breastfeeding	2,023 (77.0)	234 (82.4)		
Artificial feeding	36 (1.4)	7 (2.5)		
Mixed feeding	566 (21.6)	43 (15.1)		
Cesarean section (*n*, %)	1,279 (48.7)	120 (42.3)	4.298 b	0.038
Whether used antibiotics in the infant (*n*, %)	211 (8.0)	134 (47.1)	375.670 b	<0.001
Smoking (*n*, %)	255 (9.7)	43 (15.1)	8.208 b	0.004
keeping pets (*n*, %)	848 (32.3)	74 (26.1)	4.622 b	0.032
Incidence of food allergy in 18 months children	118 (4.5)	23 (8.1)	7.215 b	0.007
Incidence of food allergy in 3 years children (*n*, %)	183 (7.0)	26 (9.2)	2.460 b	0.117

BMI, body mass index; GBS, group B Streptococcus; IAP, intrapartum antibiotic prophylaxis a represents for t test and b represents for chi-square test.

### Characteristics of GBS-IAP

The results in Table 2 revealed that all mothers in the GBS-IAP group received specific GBS-IAP treatment, and 30 mothers (10.6%) received additional antibiotics. In the without GBS-IAP group, 77 (2.9%) mothers received specific GBS-IAP treatment, and 1,634 (62.2%) received additional antibiotics. 956 (36.4%) did not receive any antibiotics. Compared with mothers in the without GBS-IAP group, mothers in the GBS-IAP group received more penicillin (74.3% vs. 62.3%) and less ampicillin (18.3% vs. 26.0%) in specific GBS-IAP antibiotics, and the duration of treatment with specific GBS antibiotic was longer (median 11.22 h vs. 2.56 h). Among the additional use of other antibiotics, the with GBS-IAP group received the highest proportion of Piperacillin tazobactam (76.7%), while the without GBS-IAP group had a higher proportion of Cefoxitin use (74.2%). Women in the with GBS-IAP group received longer additional antibiotic treatment than women in the without GBS-IAP group (median 3.48 h vs. 0.91 h). Besides, the antibiotics use of emergency and planned caesarean sections in GBS-IAP group and without GBS-IAP group was presented in the Supplementary Table S4.

**Table 2:** Characteristics of GBS-IAP between without GBS-IAP group and with GBS-IAP group.

Characteristics	GBS-IAP	without GBS-IAP
	*n* = 284	*n* = 2625
Type of GBS-speciﬁc antibiotic, *n* (%)		77 (2.9)
Ampicillin	52 (18.3)	20 (26.0)
Cefazolin	21 (7.4)	9 (11.7)
Penicillin	211 (74.3)	48 (62.3)
Hours from ﬁrst antibiotic dose to delivery, median (IQR)	11.22 (7.42–16.54)	2.56 (1.76–3.58)
Exposure to other antibiotic (not GBS-specific) before delivery, *n* (%)	30 (10.6)	1,634 (62.2)
Clindamycin	3 (10.0)	103 (6.3)
Piperacillin tazobactam	23 (76.7)	210 (12.9)
Azithromycin or erythromycin	3 (10.0)	22 (1.3)
Ceftriaxone	1 (3.3)	87 (5.3)
Cefoxitin	0	1,212 (74.2)
Hours from first dose of other antibiotic to delivery, median (IQR)	3.48 (1.49–6.48)	0.91 (0.51–1.81)

### Incidence of food allergy in children

Children were followed up to 18 months old, and 23 children suffered from food allergy in the group with GBS-IAP and 118 in the group without GBS-IAP. And the group with GBS-IAP showed a significantly higher incidence than the group without GBS-IAP (8.1% vs. 4.5%).

Then children were followed up to 3 years old, and a total of 209 children suffering from food allergy, with 26 in the group with GBS-IAP and 183 in the group without GBS-IAP. The incidence of food allergy in the group with GBS-IAP was higher than in a group without GBS-IAP (9.2% vs. 7.0%), but the difference was not statistically significant (*P* = 0.146).

As shown in Figure 2, Kaplan-Meier survival analysis results demonstrated that the cumulative incidence of food allergy in the group with GBS-IAP was higher than that in a group without GBS-IAP in children aged less than 18 months (*P* = 0.005), but no significant differences were observed in children aged less than three years (*P* = 0.146).

### The effect of maternal IAP on food allergy in children

Table 3 presented the univariate and multivariate associations between maternal GBS-IAP and food allergy in children. The univariate cox proportional hazards model in children aged less than 18 months revealed that children in the GBS-IAP group had faster development and increased risk of food allergy when compared with children in the group without GBS-IAP (H.R.: 1.887,95% CI: 1.207–2.950, *P* = 0.005), so was the multivariate model (H.R.: 1.906,95% CI: 1.158–3.137, *P* = 0.011). Nevertheless, both univariate (HR: 1.343, 95%CI: 0.891∼2.026, *P* = 0.159) and multivariate (HR: 1.253, 95%CI: 0.796∼1.972, *P* = 0.329) cox proportional hazards model in children aged less than three years showed no significant differences between children in the group with GBS-IAP and group without GBS-IAP.

**Table 3:** Associations between intrapartum antibiotic prophylaxis (IAP) and risk of food allergy.

	Variables	18 months		36 months	
		H.R. (95% CI)	*P*	H.R. (95% CI)	*P*
Model 1	Without GBS- IAP	1[Reference]		1[Reference]	
	With GBS-IAP	1.887 (1.207∼2.950)	0.005	1.343 (0.891∼2.026)	0.159
Model 2	Without GBS-IAP	1[Reference]		1[Reference]	
	With GBS-IAP	1.906 (1.158∼3.137)	0.011	1.253 (0.796∼1.972)	0.329
	Mothers age	0.985 (0.942-1.030)	0.503	0.988 (0.948-1.026)	0.532
	Breastfeeding	1[Reference]	0.670	1[Reference]	0.570
	Artificial feeding	0.759 (0.186-3.103)	0.701	0.434 (0.107-1.762)	0.434
	Mixed feeding	1.383 (0.941-2.034)	0.099	1.205 (0.875-1.660)	0.253
	Weight of birth	1 (1.000-1.001)	0.313	1 (1.000-1.000)	0.400
	Mothers without allergic history	1[Reference]		1[Reference]	
	Mothers with allergic history	5.618 (3.921-8.050)	<0.001	5.150 (3.811-6.960)	<0.001
	One child	1[Reference]		1[Reference]	
	The second child and more	0.783 (0.538-1.138)	0.199	0.736 (0.539-1.006)	0.084
	Vaginal delivery	1[Reference]		1[Reference]	
	Cesarean section	0.604 (0.424-0.859)	0.005	0.626 (0.469-0.836)	0.002
	Gestational age	0.780 (0.666-0.913)	0.002	0.751 (0.659-0.855)	<0.001
	Female	1[Reference]		1[Reference]	
	Male	0.974 (0.694-1.368)	0.881	1.064 (0.807-1.403)	0.661
	No antibiotics are used after birth	1[Reference]		1[Reference]	
	Antibiotics used after birth	0.871 (0.515-1.474)	0.606	1.152 (0.759-1.747)	0.507
	BMI	0.931 (0.843-1.029)	0.162	0.995 (0.919-1.078)	0.804
	No smoking	1[Reference]		1[Reference]	
	Smoking	1.644 (0.959-2.817)	0.071	1.262 (0.761-2.092)	0.702
	Without human pets	1[Reference]		1[Reference]	
	keeping pets	1.140 (0.800-1.624)	0.470	1.246 (0.934-1.660)	0.098

^a^ GBS, group B Streptococcus; IAP, intrapartum antibiotic prophylaxis. Model 1 represents the Unadjusted Cox proportional hazards regression model on food allergy, and Model 2 represents the Adjusted Cox proportional hazards regression model on food allergy. BMI represents Maternal pre-pregnancy BMI.

**Figure 3:**
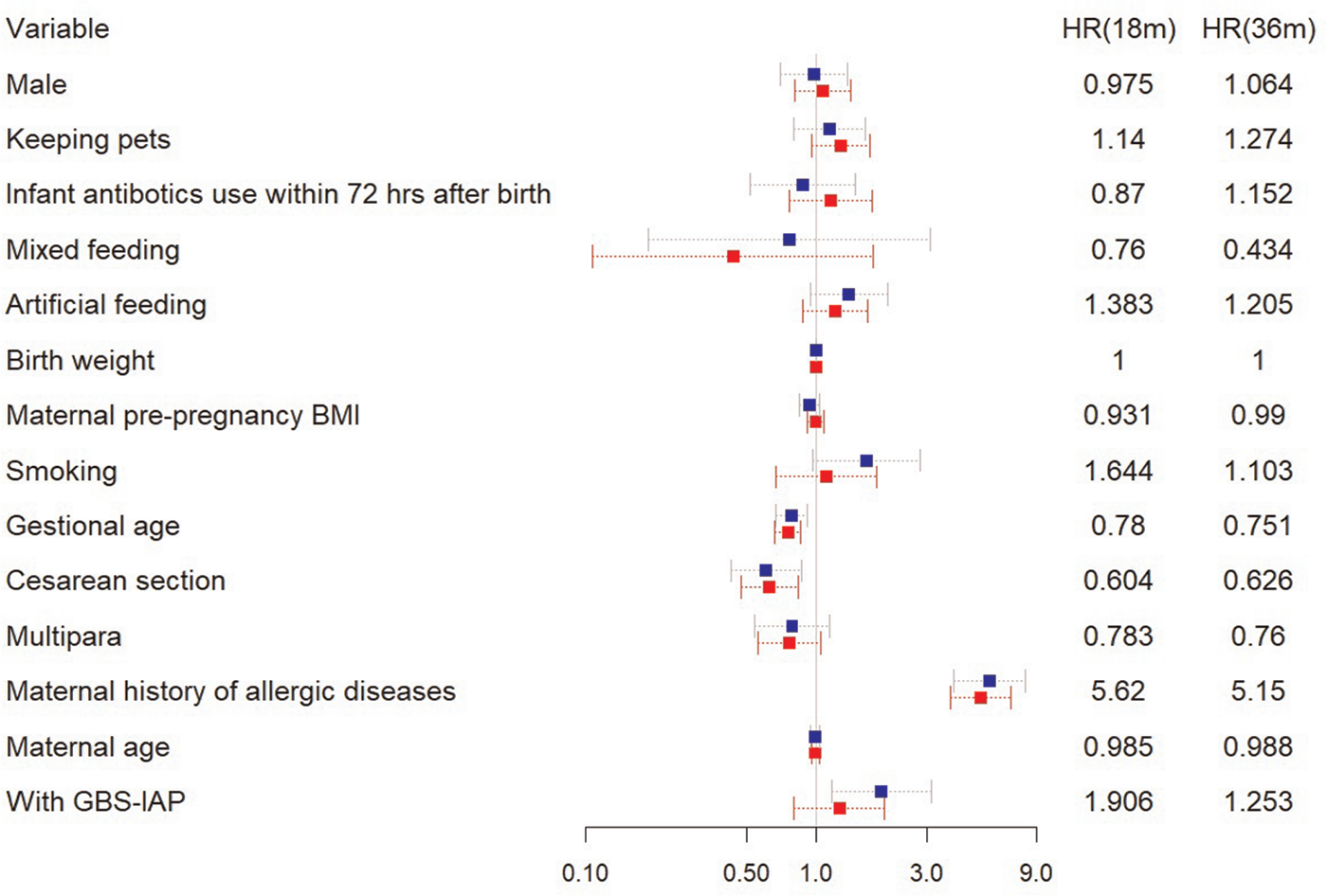
Forest plot for multivariate Cox proportional hazards regression analysis of outcomes. HR, hazard ration; GBS, group B Streptococcus; IAP, intrapartum antibiotic prophylaxis; BMI, body mass index. Blue square represents a follow-up of 18 months. and red symbol represents followed-up to 3 years old.

As in Figure 3, it was also found that maternal history of allergic diseases (H.R.: 5.150, 95% CI: 3.811–6.960) was associated with an elevated risk of food allergy in children under three years old which may accelerate the development of food allergy. Some factors, including the cesarean section (HR:0.626,95% CI:0.469–0.836) (actually only planned caesarean section, see Supplementary Table S6) and larger gestational age (HR: 0.751,95% CI:0.659–0.855), were also associated with the risk of food allergy in children.

## Discussions

Food allergies generally occur in infants and young children, and this study found an incidence of approximately 7.2% in Taixing, which is relatively consistent with previous studies (5). Meanwhile, we discovered that IAP of GBS may increase the risk of food allergy in children early after birth (18 months ago), but it has no significant effect on the risk of food allergy in children aged three years old.

Plenty of studies focused on the effect of early-life antibiotic use on food allergy in children, but studies addressing the association between prenatal antibiotic use and food allergy in children were relatively few, and the results were sometimes controversial. For example, a birth cohort study from Europe in 2019 found a positive association between prenatal antibiotics use and food allergy in the first year of life in rural children (22), and so was a prospective birth cohort from China (12). However, no such relationship was demonstrated in another case-control study in the U.S (18).

Previous studies generally had shortcomings, such as confounding factors not being measured (e.g., family history of food allergy, history of environmental exposure, etc.). Moreover, using ICD-9-CM codes to identify food allergies may lead to misclassifying true IgE-mediated food allergies. In addition, these studies have focused on early childhood antibiotic use in relation to the risk of food allergy, but few studies examined the relationship between GBS-IAP as a specific factor and the risk of food allergy in children. Therefore, the present study was conducted to further validate the association between GBS-IAP and food allergy.

The actual cause of food allergies is under debate, but it is widely accepted that altered diversity in gut bacterial levels is the main reason for the increased incidence of food allergies (23). Several landmark studies over the past few years have reported microbiome characteristics in food allergy, particularly in children, suggesting that microecological dysregulation in early life may predict food allergy. Besides, early colonization of the infant gut microbiota has been proven to influence the risk of food allergies later in life.

Intrapartum antibiotic prophylaxis (IAP) is currently recommended to prevent early-onset neonatal illness caused by Group B Streptococcus (GBS), resulting in 25%–30% of U.S. women and their newborns being exposed to intrapartum antibiotics. It had been shown (24) that microbiota development in term infants with a vaginal delivery was affected over six months after being exposed to IAP. Specifically, IAP first alters the number of microorganisms colonizing the gut, leading to a reduction in commensal bacteria and the persistence of potentially pathogenic bacteria. In particular, the abundance of bifidobacteria was reduced, while there was a significant increase in the quantity of E. coli. Although the gut flora of infants gradually recovers over time after being disrupted, it may recover inappropriately or incompletely, and the full significance of this is not known (25). Thus, a transient state of ecological dysbiosis occurs in the developing gut microbiota of infants (26), which may also be related to the development of allergic disease after birth in children. Similarly, we found that the cumulative incidence of food allergy was higher and developed more rapidly in children exposed to IAP at the follow-up to 18 months, with no difference in the cumulative incidence of food allergy by three years. It also suggested that the effect of IAP gradually disappears as the children's gut flora matures.

In this study, we found that compared with women in the without GBS-IAP group, penicillin was the most commonly used antibiotics in the with GBS-IAP group. Antibiotics for IAP prophylaxis, although usually narrow-spectrum (e.g., penicillin) and relatively short-lived, lead to lasting changes in the composition and diversity of the microbiota. In 2017, Nogacka et al. examined the design of the gut microbiota in infants with a history of penicillin IAP treatment during the first three months of life (27), and they found that not only did the number of commensal bacteria decrease more than a one-time point but also the abundance of potentially pathogenic bacteria increased 90 days after penicillin IAP intervention. In this respect, the findings of Taipianen et al. were particularly noteworthy, as their study suggested that the effects of IAP on ecological dysbiosis of the gut composition in infants may be comparable to postnatal antibiotic exposure (28).

With the limitation in manuscript, we did not include clinical manifestations and food types in the main text but presented them in the Supplementary Tables S1, S2, so was the characteristics of children born to mothers with allergic diseases history between with GBS-IAP group and Without GBS- IAP group in Supplementary Table S3.

Besides, we found that women in the with GBS-IAP group had longer duration of antibiotics use when compared with women in the without GBS-IAP group. It was previously believed that the fetus was in a sterile environment before delivery, and colonization of the neonatal gut flora tended to occur after birth. However, recent studies have found that colonization of the intestinal flora begins *in utero* (29), which means that colonization initially starts before birth. Besides, the timing of the IAP intervention is implemented 4 h before delivery, which overlaps with the establishment of the first batch of colonized gut microbes in the newborn (30). The transmission of the mother-infant microbiome is part of the anticipation of labor. Therefore, blocking this transmission during this critical developmental period may alter the immune initiation associated with early microbial colonization (31).

Plenty of studies have reported that prenatal antibiotic exposure was associated with food allergy in offspring (12, 22). Nevertheless, fewer studies have revealed the effect of the duration of antibiotic exposure on the food allergy in children. We assume that there is a biological plausible relationship between antibiotics and immune changes in early life, and treatment time may be an important determinant, which is worthy of research and explorations in the future.

According to previous studies, the development of food allergy is the result of a multifactorial interaction, particularly closely related to genetic factors and environmental factors. The story of allergy is significantly related to genetic factors, with the risk of a child developing food allergy increasing up to twofold when only one parent has a history of allergic disease. Nevertheless, the risk increased three times when both parents had a history of allergic disease (32). In addition to specific allergy risk genotypes, it has been suggested that the associations between family history of food allergy and risk in infancy may be due to reverse causality related to long-term avoidant or delayed intake of specific foods. Our study also confirmed that maternal history of allergic diseases was a significant independent risk factor for food allergy in children.

Few studies have shown that cesarean section is a protective factor in the occurrence of allergic diseases. Previous studies have found the differences in the composition of intestinal microbiota between infants born by cesarean section and those born by vaginal delivery, which may explain the differences in the risk of allergic diseases (33–35). If the relationship between the mode of delivery and the risk of allergic diseases could be explained by the microbiome-seeding hypothesis, then cesarean section births would be expected to be associated with allergies regardless of indication (i.e., elective vs. emergency). However, previous cohort studies have found the difference between selective cesarean section and cesarean section of developing allergic diseases, which indicated that the relationship between cesarean section and allergic diseases is affected by the indication (36, 37).

Similar to our results, a prospective study in Finland have found the significantly less allergic sensitization among children delivered by cesarean section when compared with children delivered by vaginal delivery, and allergic sensitization tended to be more common in children with longer duration of labor before birth (38). Moreover, a nested case-control trial in Japan revealed that vaginal delivery was significantly associated with an increased risk of food allergy in infancy, which may be due the stress and inflammatory stimulation caused by longer delivery period (21).Meanwhile, it was reported that only emergency caesarean delivery was significantly associated with doctor-diagnosed food allergy In the Upstate KIDS cohort (36).

Collier et al. reported that exposure to labour, as compared to delivery by prelabour Caesarean section, was associated with a transient decrease nTreg, which affected prenatal immune development and led to the increased risk of food allergy in childhood (39). These studies and assumptions may be the explanations of our study, that is, a longer period of delivery may increase the risk of food allergy in infancy by stimulating inflammatory pathways, which deserves further in-depth study in the future.

To explore the potential roles of labour, we additionally categorized cesarean section into emergency cesarean section and planned cesarean section. Meanwhile, the incidence of food allergy and the association between delivery mode and food allergy were described in Supplementary Tables S5, S6, in which only planned cesarean delivery was shown to be related with reduced food allergy risk.

There are some limitations to this study. First, this study was a retrospective cohort, and much of the information relies on parental memories, which may cause bias and reduce the credibility of the results. Then, this study lacked detailed information on the paternal history of allergy, paternal smoking history, family income and maternal antibiotic use during pregnancy. In the future, we intend to conduct a large sample prospective cohort, where stools of newborns delivered after IAP intervention are collected simultaneously to examine the difference in flora diversity and enrichment with high-throughput sequencing. Besides, a longitudinal study will be conducted to investigate the impact of IAP on various health outcomes in children to assess the risk and benefit of GBS prevention strategies accurately.

## Data Availability

The original contributions presented in the study are included in the article/**Supplementary Material**, further inquiries can be directed to the corresponding author/s.
