# Supplementary material for: Study on the relationship between intrapartum group B streptococcus prophylaxis and food allergy in children
Source: Front Pediatr. 2022 Dec 2;10:1039900. doi: 10.3389/fped.2022.1039900 (PMC9755746; doi:10.3389/fped.2022.1039900)
Supplement: Supplementary file 1 [file Datasheet1.pdf]

Supplemental Table 1 Clinical manifestations in children at the age of 18 months and 3 years

Clinical manifestations*	18m (n=141)	36m (n=129)
abdominal pain, diarrhea or hematochezia	92	61
feeding difficulty	41	13
rash with pruritus	40	81
sneezing, nasal congestion and runny nose	13	21
Red eyes and tears	8	13
Itching, tingling or swelling of the mouth, lips or throat	7	11
dyspnea	4	5

* Multiple clinical manifestations could be observed in one child.

Supplemental Table 2 Food types causing food allergy in children at the age of 18 months and 3 years

Food type	18m (n=141)	36m (n=129)
Milk	72	34
Eggs	24	36
Wheat	13	19
Fish and shrimp	10	13
Pork	5	5
Peanut	5	8
Mango	4	5
Milk and eggs	6	4
Milk and wheat	2	2
Fish, shrimp and wheat	0	2
Fish, shrimp and mango	0	1

Supplemental Table 3 Characteristics of children born to mothers with allergic diseases history between with GBS-IAP group and Without GBS- IAP group

Characteristic	Without GBS- IAP (n=225)	With GBS- IAP (n=39)	*t*/χ^2	*P value*
*Mother's age (years)	27.50±4.07	28.18±4.41	-0.954	0.341
*Mother's BMI（x±s）	20.84±1.80	20.60±1.30	0.813	0.417
**Parity （n，%）			0.936	0.333
One child	134（59.6）	20（51.3）		
**Second child or more	91（40.4）	19（48.7）		
**GBS Screening（n，%）			233.186	<0.001

Positive	2(0.9)	37(94.9)		
Negative	2527(96.3)	14(4.9)		
**Whether received higher education（n，%）	176(78.2)	30(76.9)	0.033	0.856
**Gender （man，n，%)	126（56.0）	16（41.0）	2.998	0.083
*Gestational age（weeks x±s）	39.3±1.1	38.8±1.1	2.596	0.010
*Weight of birth （g x±s ）	3388±399	3348±380	0.605	0.548
**Feeding method（n，%）			1.761	0.358
Breastfeeding	155（68.9）	31（79.5）		
Artificial feeding	7（3.1）	1（2.6）		
Mixed feeding	63（28.0）	7（17.9）		
**Cesarean section（n，%）	105（46.7）	15（38.5）	0.903	0.342
**Whether used antibiotics in the infant （n，%）	18（8.0）	20（51.3）	50.533	<0.001
Smoking	1(0.4)	19(48.7)	103.823	<0.001
keeping pets	116(51.6)	17(43.6)	0.844	0.358
**Incidence of food allergy in 18 months children	31(13.8)	19(48.7)	26.431	<0.001
**Incidence of food allergy in 3 years children（n，%）	48（21.3）	21（53.8）	18.200	<0.001

Supplemental Table 4 Characteristics of GBS-IAP between without GBS-IAP group and with GBS-IAP group

Characteristics	GBS-IAP	without GBS-IAP
	n = 284	n = 2625
Type of GBS-specific antibiotic,n (%)		77(2.9)
Ampicillin	52(18.3)	20 (26.0)
Cefazolin	21 (7.4)	9(11.7)
Penicillin	211(74.3)	48(62.3)
Hours from first antibiotic dose to delivery, median (IQR)	11.22 (7.42–16.54)	2.56(1.76–3.58)
Exposure to other antibiotic (not GBS-specific) before delivery, n (%)	30(10.6)	1634(62.2)
Clindamycin	3(10.0)	103(6.3)
Piperacillin tazobactam	23(76.7)	210(12.9)
Azithromycin or erythromycin	3(10.0)	22(1.3)
Ceftriaxone	1(3.3)	87(5.3)
Cefoxitin	0	1212(74.2)
Hours from first dose of other antibiotic to delivery, median (IQR)	3.48 (1.49–6.48)	0.91 (0.51–1.81)
